# Crystallography of metal–organic frameworks

**DOI:** 10.1107/S2052252514020351

**Published:** 2014-10-28

**Authors:** Felipe Gándara, Thomas D. Bennett

**Affiliations:** a Department of New Architectures in Materials Chemistry, Materials Science Institute of Madrid – CSIC, Sor Juana Inés de la Cruz 3, Madrid 28049, Spain; b Department of Materials Science and Metallurgy, University of Cambridge, 27 Charles Babbage Road, Cambridge CB3 0FS, England

**Keywords:** MOFs, non-ambient crystallography, crystal growth

## Abstract

Metal–organic frameworks (MOFs) are one of the most intensely studied material types in recent times. Their networks, resulting from the formation of strong bonds between inorganic and organic building units, offer unparalled chemical diversity and pore environments of growing complexity. Therefore, advances in single-crystal X-ray diffraction equipment and techniques are required to characterize materials with increasingly larger surface areas, and more complex linkers. In addition, whilst structure solution from powder diffraction data is possible, the area is much less populated and we detail the current efforts going on here. We also review the growing number of reports on diffraction under non-ambient conditions, including the response of MOF structures to very high pressures. Such experiments are important due to the expected presence of stresses in proposed applications of MOFs – evidence suggesting rich and complex behaviour. Given the entwined and inseparable nature of their structure, properties and applications, it is essential that the field of structural elucidation is able to continue growing and advancing, so as not to provide a rate-limiting step on characterization of their properties and incorporation into devices and applications. This review has been prepared with this in mind.

## Abstract 2

Recent advances in the crystallography of metal–organic frameworks (MOFs) are reviewed, including crystal growth, structural elucidation, *in-situ* and non-ambient crystallography.

## 1. Introduction   

Metal–organic frameworks (MOFs) are a class of materials constructed by the combination of organic linkers and metal ions to produce extended networks that possess pores and cavities of adjustable size and shape (Furukawa *et al.*, 2013 ▶). MOFs are among the most porous materials known, some of them possessing BET and Langmuir specific surface area values higher than 6000 m^2^ g^−1^ and 10000 m^2^ g^−1^, respectively (Furukawa *et al.*, 2010 ▶; Farha *et al.*, 2012 ▶). MOFs are becoming increasingly important because of their applications in many fields, such as gas storage (Mason *et al.*, 2014 ▶), gas separation (Britt *et al.*, 2009 ▶; McDonald *et al.*, 2012 ▶), catalysis (Phan *et al.*, 2011 ▶), thermal conversion (Khutia *et al.*, 2013 ▶), drug delivery (Cunha *et al.*, 2013 ▶), harmful substance storage (Bennett *et al.*, 2013 ▶) and biomedical imaging.

The ease with which the constituents of MOFs can be varied and functionalized has led to the synthesis of a large number of new materials. The ultimate goal of a significant segment of research is the creation of completely new pore environments having increasingly complex chemical components and structural characteristics. As a result, an enormous array of new compounds are consistently being prepared and reported in the scientific literature, which necessitates the structural characterization of frameworks of progressively increasing complexity. The crystal structures of MOFs are, in general, unambiguously determined using crystallographic techniques such as X-ray diffraction, the availability of which is undoubtedly another factor contributing to the popularity of the MOF field. However, although these techniques are widely employed and well understood (being over a century old), the structure solution of highly porous materials is not always a straightforward process. Factors which prevent trivial structural elucidation include the presence of a large amount of pore-occupying, disordered solvent molecules (sometimes resulting in refined structures with residual values larger than those typically reported for molecular crystals), or difficulties associated with the growing of good diffraction-quality crystals. In addition, new techniques are being developed and applied to further study the fascinating properties of these materials, which in many cases requires diffraction under non-ambient conditions (*i.e.* high pressure, high temperature).

In this article we give a general view of some of the latest progress in crystallographic characterization being implemented in the study of MOFs.

## 2. Crystal growth and single-crystal structure analysis of MOFs   

Single-crystal X-ray diffraction is rightly considered the ultimate technique for the structural determination of crystalline materials. This is a very mature technique that has become readily accessible, and this accessibility is accompanied by vast improvements in the related instrumentation. The recent development of bright microfocus sources combined with highly sensitive area detectors allows for single-crystal data collection to be routinely performed in laboratories within a few hours, even with microsized crystals.

Keeping this in mind, the success of a diffraction experiment depends almost entirely on the quality of the studied specimen. It is therefore surprising that only a relatively small number of studies dedicated to the MOF crystallization process exist (Attfield & Cubillas, 2012 ▶; Guo *et al.*, 2012 ▶; Hermes *et al.*, 2007 ▶). Of these, the majority of studies are focused on obtaining nanosized crystals of already-known materials and on the control of their size and morphology. Comparatively few reports exist on the optimization of the crystal growing process, with the aim of obtaining large single crystals of MOFs. In contrast to molecular crystals (recrystallized from solution until the optimal growing conditions are found), MOF crystal growth takes place during the synthesis reaction for extended structures. Synthetic conditions must therefore be optimized not only for obtaining the desired phase, but also to control crystallization kinetics in order to improve the crystal size and quality. Small variations in the synthetic conditions have been observed to influence crystallization, but may also result in the appearance of other polymorphic phases. Combined experimental and computational studies are being used to identify the driving forces in the formation of these different crystal phases during the solvothermal synthesis of MOFs (Platero-Prats *et al.*, 2012 ▶).

**Figure 1:**
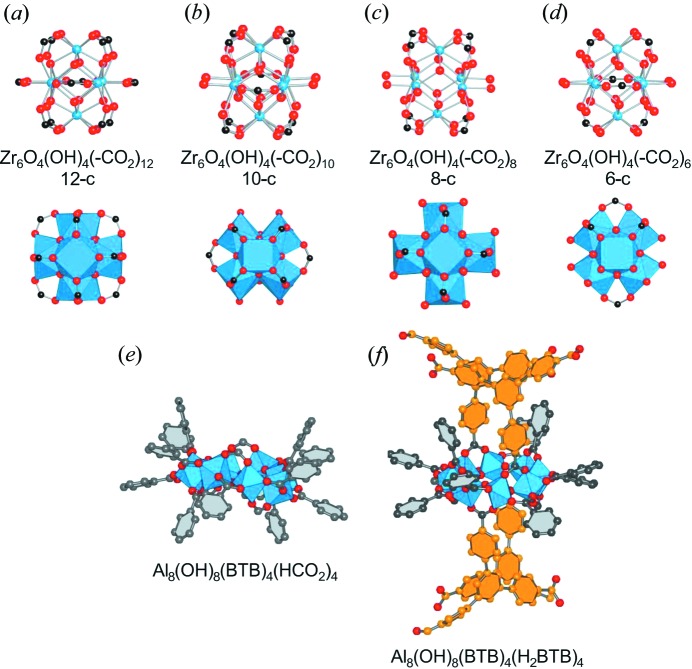
Inorganic SBUs (Secondary Building Units) of various coordination numbers are known in the family of the zirconium-based MOFs (*a*–*d*). The SBUs of MOF-520 (*e*) and MOF-519 (*f*) have the same number of framework BTB linkers (represented in grey), however, in the former, formate ligands are incorporated while additional BTB ligands (represented in orange) are found in the latter. Blue balls and polyhedra represent the metal atoms (Zr in *a*–*d*, Al in *e* and *f*), black and orange balls are C atoms, and red balls are O atoms.

It has been shown that the addition of modulating ligands during MOF synthesis can be used to control crystal size and shape. Typically, the modulating agent is a monocarboxylate molecule, such as acetic, benzoic or formic acid, which competes with the polycarboxylate framework-forming linkers and results in differences in crystal size and morphology (Tsuruoka *et al.*, 2009 ▶; Umemura *et al.*, 2011 ▶). This approach has been used to control the morphology of different MOF crystals in the nanometer scale, whilst it has also been reported that the presence of modulating molecules during the synthesis of the zirconium-based MOF UiO-66 [Zr_6_O_4_(OH)_4_(BDC)_6_, BDC = 1,4-benzenedicarboxylate] (Cavka *et al.*, 2008 ▶) facilitates larger crystal formation (Schaate *et al.*, 2011 ▶). In the latter, the authors proposed that the formation of complexes between the Zr anions and the modulating agent benzoic acid slows down the formation of MOF crystal nuclei. In addition to the variation of crystal size, the presence of modulating monocarboxylate agents might also influence the structure of the framework. In some cases the modulator is effectively incorporated into the network structure through replacement of the framework linkers, which creates connectivity defects in the structure (Wu *et al.*, 2013 ▶). The presence of these defects results in differences in the sorption properties of those MOFs. UiO-66 samples prepared with different amounts of acetic acid as the modulating agent displayed pore volumes ranging from 0.44 to 1.0 cm^3^ g^−1^. This was similarly observed for the zirconium fumarate (Wissmann *et al.*, 2012 ▶) MOF-801 [Zr_6_O_4_(OH)_4_(CO_2_–C_2_H_2_–CO_2_)_6_], which displays different gas and water sorption properties depending on the amount of formic acid employed during the synthesis (Furukawa *et al.*, 2014 ▶). Incorporation of the modulating agent without the introduction of structural defects is also possible, resulting in SBUs (Secondary Building Units) of various coordination number and different framework topologies. Thus, several zirconium-based MOFs have been reported having SBUs with coordination number 12, 10, 8 or 6 (Figs. 1 ▶ *a*–*d*). Moreover, the overall framework connectivity can be maintained while incorporating the modulators into the SBUs, as shown by the aluminium-based compounds MOF-519, [Al_8_(OH)_8_(BTB)_4_(H_2_BTB)_4_] and MOF-520, [Al_8_(OH)_8_(BTB)_4_(HCO_2_)_4_], which are constructed using the tritopic linker benzenetribenzoic acid (H_3_BTB) and possess the same SBU type and overall network topology. MOF-520, prepared in the presence of formic acid, contains an inorganic SBU with four aluminium-coordinated formate ligands without impact on framework connectivity (Fig. 1 ▶ *e*). In contrast, these sites are occupied by four additional monocoordinated H_2_BTB ligands in MOF-519 (where there is no addition of extra carboxylic acid species in the synthesis) (Fig. 1 ▶ *f*), which dangle into the pores and drastically modify the sorption properties of this material which exhibits a high volumetric methane uptake (Gándara *et al.*, 2014 ▶). Related SBUs are known for other aluminium-based MOFs of the CAU series (Ahnfeldt *et al.*, 2009 ▶). These MOFs are prepared in the absence of modulating agents, and instead of monocarboxylate ligands it is alkoxide species derived from the synthetic solvent which are incorporated into the SBUs.

## 3. Structure solution from powder diffraction data   

When MOF single-crystal growth is not possible, structure solution from powder diffraction data can be accomplished, although this is usually a more challenging process. So far, several different standalone or combined approaches have been successfully employed to solve the crystal structure of MOFs using powder diffraction.

**Figure 2:**
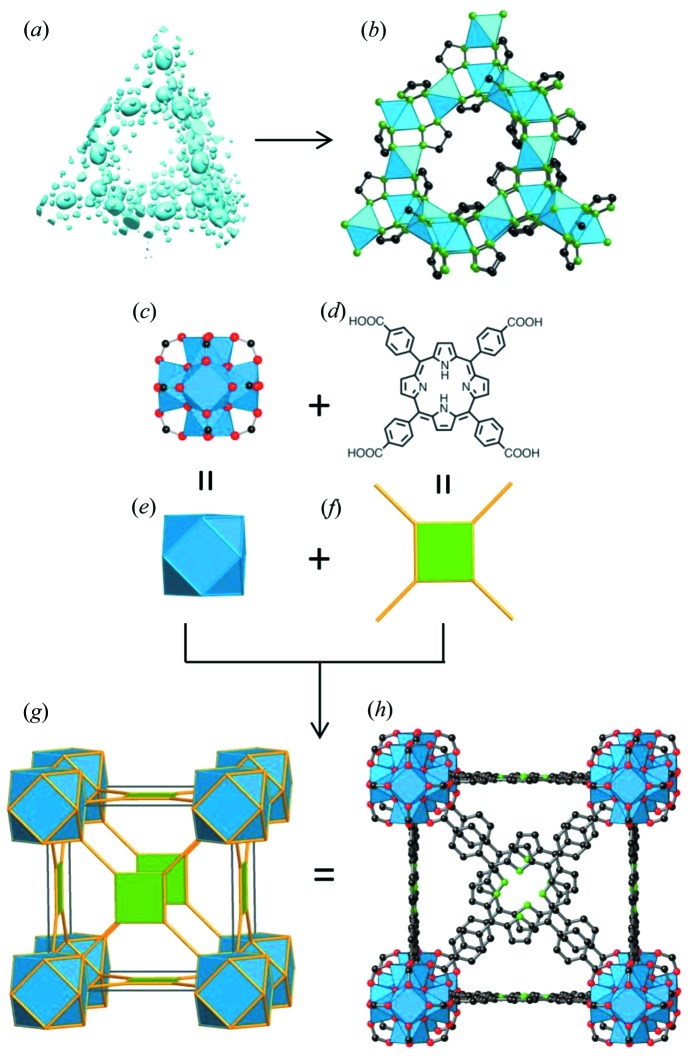
Structure elucidation from PXRD data analysis: The application of the charge-flipping method to PXRD data resulted in an electron density map (*a*) from which the structure of metal-triazolates, METs (*b*), was obtained. Following the reticular approach, the 12-connected zirconium SBU (*c*) and the tetracarboxyphenylporphyrin organic linker (*d*) are simplified to a cuboctahedron (*e*) and a square (*f*), respectively. Their combination produces the edge transitive net **ftw** (*g*), from which the structure of MOF-525 (*h*) is directly derived.

The structural solution of MOFs without any previous information has been reported using the application of direct methods to powder data, notably in the case of UiO-66 (Cavka *et al.*, 2008 ▶), which was solved by direct methods implemented in the program *EXPO* (Altomare *et al.*, 2013 ▶) using high-quality data collected with synchrotron radiation. The process is not dissimilar to that carried out for any other type of material, with a typical approach involving the pattern indexing, intensity integration, structure solution and final Rietveld refinement. This same process can be carried out with the charge-flipping method (Oszlányi & Sütő, 2008 ▶) instead of direct methods, as in the case of another family of MOFs known as metal-triazolates (METs) (Figs. 2 ▶ *a* and *b*) (Gándara *et al.*, 2012 ▶). The procedure to obtain the initial solution is equivalent to the one that would be followed with single-crystal diffraction data, although includes modifications such as histogram matching with chemical composition (Baerlocher *et al.*, 2007 ▶). Recently it has also been reported that the solution obtained with application of the charge-flipping method to powder diffraction data can be greatly improved, provided that an initial set of phases is obtained for at least low-resolution reflections. These initial sets are supplied to the input data so that they are not randomly assigned in the first step of the charge-flipping cycle. This set of phases can be obtained with the use of high-resolution electron microscopy (Sun *et al.*, 2009 ▶), but most interestingly it has been recently demonstrated that they can also be obtained with the use of only powder X-ray diffraction (PXRD) data, by applying the charge-flipping method initially to only a few subsets of reflections corresponding to low-resolution two-dimensional projections (Xie, McCusker *et al.*, 2011 ▶; Xie, Baerlocher *et al.*, 2011 ▶). The use of these phases obtained by either method in the subsequent application of the charge-flipping method with the full data set resulted in greatly improved electron density maps that could be interpreted, unveiling the structure of microporous zeolites. It can be anticipated that this same method can be applied to the structure solution of porous MOFs with only the use of powder diffraction data.

### 3.1. Powder data and the topological approach to crystal structure solution   

When an *ab initio* structure solution cannot be achieved, structure solution from powder diffraction data can also be accomplished with direct space solution methods, provided that structural information about the components of the crystal is known. In the case of MOFs, this condition is fulfilled with the use of rigid organic linkers, and global optimization methods have been successfully employed to solve the structure of MOFs (Masciocchi *et al.*, 2010 ▶). While no assumption about framework topology or connectivity of the framework is used in the case of molecular crystals, such information greatly aids crystal structure solution of MOFs. Reticular chemistry (Ockwig *et al.*, 2005 ▶; O’Keeffe & Yaghi, 2005 ▶, 2012 ▶) has demonstrated that some networks can be preferentially obtained with the use of specific building units. Although in principle there are an infinite number of possible combinations of different sub-units in extended networks, it has been observed that only a small number of them have been realised – in particular those of higher symmetry. For example, when a framework is formed by the combination of two different types of nodes, edge-transitive networks (Delgado-Friedrichs *et al.*, 2006 ▶) (meaning that all the edges in the structure are equivalent) are the most frequently observed. Thus, it is possible to limit to some extent the number of networks that can be constructed by the combination of building blocks with the selected geometries (Delgado-Friedrichs *et al.*, 2006 ▶). Consequently, crystal structure models are made according to the most feasible topology that can be obtained with the employed building blocks. In practical terms, the process of the construction of a computer model consists of the replacement of the edges and nodes of the underlying network by the atoms of the employed molecules. The coordinates of the edges and nodes for the different types of networks can be obtained from different databases. The Reticular Chemistry Structure Resource (RCSR) (O’Keeffe *et al.*, 2008 ▶) contains information on networks with corresponding vertices and edge coordinates in their maximum symmetry embedding. If the actual symmetry of the molecules is lower than that of the ideal network, a model can be built in a subgroup of the original space group. For example, the computer program *TOPOS* (Blatov *et al.*, 2014 ▶) contains information from different databases, including the RCSR, and it can generate a file in a crystallographic format that can be modified accordingly with the employed chemical units. In addition, this program contains a symmetry module with the group–subgroup relationships to transform the atomic coordinates from a given space group to a different one, which makes it very useful to obtain the fractional coordinates of the desired network in a lower symmetry space group.

In addition to the building of models from ideal networks, crystal models related to existing compounds have also been employed. Particularly, isoreticular expansion of MOFs results in compounds that have the same underlying topology, but different metrics. The current record of isoreticular expansion has been attained with the IRMOF-74 series (Deng *et al.*, 2012 ▶), where the same structure type can be obtained by expanding the length of the linker from the one phenylene ring present in the original MOF-74 (Rosi *et al.*, 2005 ▶) [*M*_2_DOT, DOT = dioxidoterephthalate, *M* = Zn (Rosi *et al.*, 2005 ▶), Mg (Dietzel *et al.*, 2008 ▶), Co (Dietzel *et al.*, 2005 ▶), Ni (Dietzel *et al.*, 2006 ▶), Fe (Bhattacharjee *et al.*, 2010 ▶), Mn (Zhou *et al.*, 2008 ▶), Cu (Sanz *et al.*, 2013 ▶) and a combination of them (Wang *et al.*, 2014 ▶)] to an upper limit of 11 phenylene rings. All the members of this series have the same topology, resulting from the rod-shaped SBU characteristic of this material. Crystal models were constructed by adding extra phenylene rings and the corresponding substituent groups that were introduced to enhance the solubility of the linkers. Rietveld refinements were performed with most of the members of the series using diffraction collected with a synchrotron source. However, the members of the series with the longest linkers resulted in powder diffraction patterns with only a very limited number of observable diffraction lines. Difficulties emerged considering that the largest member of the series has up to 85% calculated void space, resulting in structures with only a few intense diffraction lines. In the case of larger members of the series, namely IRMOF-74-IX and XI, all *hkl* reflections with *l* not equal to zero have a relative intensity below 0.3% according to the diffraction pattern calculated from the crystal model (even in the hypothetical case of a perfect crystal), which can hardly be observed in the experimental PXRD pattern. Although this results in a lack of information along the *c* axis from the experimental data, the expansion of the organic linker does not affect the chemical bonds along the SBU (composed only by metal and O atoms), and those were unambiguously determined with the smaller members of the series. In any case, the characterization of these types of compounds should be complemented with other techniques that help to confirm the proposed models, such as porosity measurements, NMR, and electron microscopy (Suga *et al.*, 2014 ▶).

Whilst the synthetic conditions leading to the formation of a given SBU have been optimized in many cases, sometimes a different SBU can be created during the MOF reaction. As previously explained, the use of modulating agents during the synthesis of zirconium-based MOFs might have an important effect in the resulting structure. This is true of MOF-525 [Zr_6_O_4_(OH)_4_(TCPP-H_2_)_3_] and MOF-545 [Zr_6_O_8_(H_2_O)_8_(TCPP-H_2_)_2_], two compounds synthesized using zirconium and a porphyrin-based linker, tetracarboxyphenylporphyrin, H_4_–TCPP–H_2_ (Morris *et al.*, 2012 ▶). By means of computer modelling and PXRD analysis, the structure of MOF-525 was found to be of the **ftw** type, which results from the combination of cuboctahedral units (the most commonly found units in zirconium based MOFs), and square units such as the porphyrin linker (Figs. 2 ▶ *c*–*h*). However, the structure of MOF-545 did not match any of the structure types based on 12-connected networks, and only after single-crystal formation was it revealed that the connectivity of the inorganic SBU differs from that of the cuboctahedral unit. The disposition of the metal atoms in the SBU remains unaltered, but part of the carboxylic acid groups are replaced by water ligands, resulting in a SBU with a lower connectivity and an overall network that is 8- and 4-connected (**csq**).

The extensive use of carboxylate-based linkers has resulted in the identification of a large number of SBUs that can be used to generate possible crystal models, a situation not yet encountered with the use of linkers with different functionalities. For example, metal-catecholates, CATs (Hmadeh *et al.*, 2012 ▶), are constructed by the linkage of metal atoms with a conjugated tricatecholate, 2,3,6,7,10,11-hexahydroxytriphenylene (H_12_C_18_O_6_, hhtp). When combining hhtp with Co or Ni, the formation of hexagonal layers was anticipated by the joining of this triangular linker through the metal atoms. After a fine tuning of the synthetic conditions by adding trace amounts of a co-solvent, it was possible to obtain a larger crystal of Co-CAT, which could be measured using synchrotron radiation. After structural solution using single-crystal diffraction data, formation of the hexagonal layers was confirmed; although it was also found that the interlayer space is occupied by discrete complexes that form a second type of layer, which is hydrogen bonded to the adjacent ones, and with the hhtp molecules stacking with a 60° rotation with respect to each other. Another member of the CAT series, Ni-CAT, was confirmed to be isostructural by means of PXRD data refinement and with the use of high-resolution microscopy it was possible to observe the hexagonal pore system and the detail of the crystal edge. Recently, a related material, Ni_3_(HITP)_2_ (HITP = 2,3,6,7,10,11-hexaiminotriphenylenesemiquinonate), has been reported with a structure consisting of slipped-parallel *AB* stacking layers (Sheberla *et al.*, 2014 ▶). The structure was solved based on the analysis of PXRD patterns, in combination with DFT calculations and X-ray absorption fine structure (EXAFS) analysis in order to propose the stacking sequence of this highly conducting compound.

## 4. Trends in MOF structural elucidation under non-ambient conditions   

The drive towards the use of MOFs in various applications has led to increasing research on their structure–property relationships (Wilmer *et al.*, 2012 ▶; Fernandez *et al.*, 2013 ▶). In particular, the proposed use of MOFs in gas sorption and separation devices, alongside the more specific areas of sensing and high-pressure chromatography (Gu *et al.*, 2012 ▶), is of concern given a relative lack of knowledge on their response to temperature, pressure and impact. Given that the properties of MOFs are intrinsically related to their porous structures, information on their structural response to these external stimuli is paramount and therefore there are several crystallographic studies of MOF gas uptake.

Crystallographic evidence and determination of gas adsorption sites were first reported for the highly porous MOF-5 [Zn_4_O(BDC)_3_] in 2005 with the use of single crystals exposed to N_2_ and Ar (Rowsell *et al.*, 2005 ▶). This study demonstrated how gas molecules are primarily adsorbed on the inorganic SBUs but also on the edges of the phenyl rings that are part of the organic linkers. A complementary neutron diffraction study was carried out to determine the position of adsorbed H_2_ molecules in this same material (Spencer *et al.*, 2006 ▶). A pair distribution function (PDF) study combining X-ray and neutron diffraction was used to investigate the interaction between adsorbed H_2_ molecules and the porous Prussian Blue analogue Mn_3_^II^[Co^III^(CN)_6_]_2_ (Chapman *et al.*, 2006 ▶). This material has a high density of accessible metal sites, which in principle could interact strongly with H_2_ molecules. Interestingly, PDF analysis shows no evidence of direct binding between accessible metal sites and the gas molecules. On the contrary, H_2_ molecules are found to be in the centre of the cavities of this material, optimizing van der Waals interactions with the framework. Neutron diffraction has proved to be particularly useful for the determination of adsorbed species in MOFs and several neutron powder diffraction studies have been used to determine the location of adsorbed guest species in the pores of MOFs, providing great insight on the host–guest interactions. The adsorption sites of CO_2_ (Queen *et al.*, 2011 ▶), O_2_ (Bloch *et al.*, 2011 ▶), CH_4_ (Wu *et al.*, 2009 ▶, 2010 ▶; Getzschmann *et al.*, 2010 ▶) or different hydrocarbons (Herm *et al.*, 2013 ▶; Bloch *et al.*, 2012 ▶) have thus been investigated.

Although crystallographic studies of MOF gas uptake and related structural deformation at relatively low pressures are reasonably well established (Carrington *et al.*, 2014 ▶), there are few reports on the use of higher pressures (> 0.5 GPa). *In-situ* X-ray powder diffraction experiments, performed in a diamond–anvil cell with a sample surrounded by a hydrostatic medium, are able to determine quickly the bulk modulus (inverse compressibility) and amorphization limits (loss of long-range order) of MOFs. Whilst sample particle size and the rate of pressure are known to modify both of the above, the nature of the pressure-transmitting fluid (PTF) used is critical – small-molecule PTFs can induce ‘hard’ regions of compressibility associated with entry into the framework cavities, before ‘softer’ regions are observed (*e.g. K*_hard_ = 118 GPa, then *K*_soft_ = 30 GPa for HKUST-1, [Cu_3_(btc)_2_(H_2_O)_2_], btc = 1,3,5-benzene-tricarboxylate) (Chapman *et al.*, 2008 ▶). Dependence on initial pore occupancy has also been observed to be crucial, *e.g.* evacuated samples of zeolitic imidazolate framework, ZIF-4 [Zn(C_3_H_3_N_2_)_2_], are significantly more compressible (*K*_0_ = 2.6 GPa) than those containing ethanol (K_0_ = 7.8 GPa) (Bennett *et al.*, 2011 ▶).

**Figure 3:**
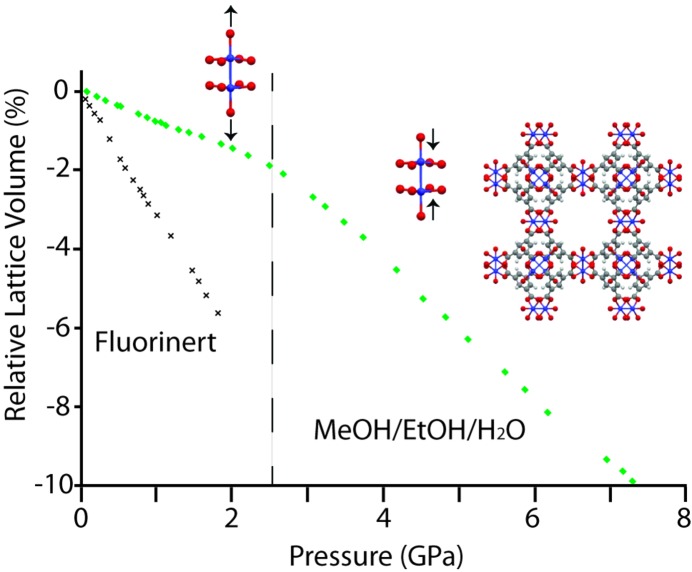
Synergy of high-pressure powder and single-crystal diffraction experiments on HKUST-1. Two regions of compressibility are noted (separated by the dotted line) in the case of small molecule pressure-transmitting fluids, which can be ascribed to initial pore filling of the framework and associated pore volume increase.

Single-crystal high-pressure experiments suffer from the detrimental effect of higher pressures upon crystal diffraction quality (particularly with large-molecule PTFs), but they are invaluable in providing accurate structural information. Notably, the entry of small molecule penetrating fluids into the pores (super-hydration) of ZIF-8 [Zn(C_4_H_5_N_2_)_2_] and MOF-5 has been observed to cause pore apertures to distort in order to accommodate an increased number of solvent molecules (gate-opening behaviour) (Moggach *et al.*, 2009 ▶; Graham *et al.*, 2011 ▶). The utility of combined single-crystal and powder diffraction studies is best illustrated with the HKUST-1 framework, in which rapid powder studies revealed multiple regions of compressibility, and slower single-crystal studies later revealed the source of a phase transition to be elongation of the compliant Cu—O axial bonds in the five-coordinate metal environment (Fig. 3 ▶) (Graham *et al.*, 2012 ▶; Chapman *et al.*, 2008 ▶). Studies on the Cu-asp (asp = aspartate) framework also revealed the flexibility of this motif (Gould *et al.*, 2012 ▶).

An emerging trend is the use of pressure to generate new MOF structures, as in the case of multiple new polymorphs of Zn(CN)_2_, or to probe the extreme mechanical flexibility of materials, *e.g.* ZAG-4, [Zn(HO_3_PC_4_H_8_PO_3_H)·2H_2_O] (Lapidus *et al.*, 2013 ▶; Gagnon *et al.*, 2013 ▶). The latter has revealed some unusual instances of negative linear compressibility (the expansion of a material along one direction on increasing hydrostatic pressure) in MOFs, which some have ascribed to the presence of a ‘wine-rack’ network motif (Li *et al.*, 2012 ▶; Cairns *et al.*, 2013 ▶). These exceptions aside, however, crystallographic evidence points to MOFs displaying the same responses to pressure as zeolites, in polyhedral tilting (Gould *et al.*, 2014 ▶), angular distortions or bond compression.

The structural response of MOFs upon heating is also of great crystallographic interest. Negative thermal expansion (the contraction of volume with increasing temperature) is of interest in MOFs, with crystallography being used to unravel sometimes extremely complicated mechanisms (Lock *et al.*, 2010 ▶). Variable-temperature powder X-ray diffraction has been used to characterize desolvation-induced phase changes, along with transitions from closed to open pore structures (‘breathing effects’) upon heating, most notably in the MIL framework family (Chen *et al.*, 2013 ▶; Zhao *et al.*, 2014 ▶).

Most recently, concerted efforts in the crystallographic community have been made to deal with disordered MOFs. Whilst collection of total scattering data and subsequent comparison between average and local framework structure has yielded useful information on disorder induced by transverse vibrations of the organic moieties in MOFs (Collings *et al.*, 2012 ▶), more extreme cases, *i.e.* amorphous MOFs (Bennett & Cheetham, 2014 ▶), push structural elucidation techniques to the limits.

Morris *et al.* applied a PDF analysis to study the structural transformation undergone by Cu-SIP3, [Cu_2_(OH)(C_8_H_3_O_7_S)(H_2_O)·2H_2_O] (Allan *et al.*, 2012 ▶). This material, formed by coordination of Cu atoms to sulfoisophthalic acid, undergoes a reversible phase transformation upon hydration/dehydration which encompasses changes in the coordination mode of the sulfonate groups and orientation of the layers that form the structure (Xiao *et al.*, 2009 ▶). During the transformation, the material loses long-range order in the temperature range 280–430 K, as shown by the absence of Bragg peaks in single-crystal X-ray diffraction experiments. A variable-temperature PDF study was carried out, allowing the proposal of a transformation mechanism that involves a change in the coordination mode of some of the sulfonate groups and the loss of coordinated water simultaneously in the same temperature step. Crystallinity is regained after all of the sulfonate groups are reoriented, and the layers that form the structure move with respect to one another. In addition, the PDF was used to show how upon adsorption of NO by the high-temperature form, the material undergoes structural changes resulting in a structure analogous to the partially dehydrated one.

Structural elucidation in this case was made possible by an exact knowledge of framework composition (the process being reversible on re-introduction of solvent), but irreversible changes are significantly harder to characterize. Examples of transitions include the metastable [(Zn*X*_2_)_3_(TPT)_2_] (*X* = I, Br, Cl, TPT = tris(4-pyridyl)triazine) series of frameworks, which upon heating convert to a thermodynamically stable, more dense product (Mart-Rujas *et al.*, 2011 ▶). Although the use of *ab-initio* structure determination allowed determination of both bounding phases, only evidence of a reconstructive transition provided any insight into the identity of the disordered intermediate.

A similar transition sequence occurs in the crystallographically less-complicated ZIF (Zeolitic Imidazolate Framework) family. Upon heating of ZIFs of composition [Zn(C_3_H_3_N_2_)_2_], solvent loss occurs without structural change, before collapse to an amorphous phase at *ca* 300°C and recrystallization at *ca* 450°C to another dense framework. PDF analysis in this case determined retention of structural correlations below 6 Å. Reverse Monte-Carlo modelling was then used to illustrate that the amorphous ZIF adopted a structure consistent with that of α-SiO_2_, although substantial constraints on metal coordination environment and bond lengths were necessary (Beake *et al.*, 2013 ▶).

Whilst non-ambient MOF crystallography is significantly more challenging due to experimental constraints or substantial disorder, development and accessibility of equipment such as the beamlines XPDF I15 at the Rutherford Appleton Laboratory or 11.3.1 at the Advanced Light Source, among many others, will prove crucial to move the field forward.

Many of the above considerations have been given to the use of crystallography and non-ambient conditions in post synthetic MOF modifications, however, a growing number of papers have been concerned with the elucidation of the mechanisms of MOF synthesis and crystallization. Specialist reaction vessels developed in house have enabled *in-situ* synchrotron powder diffraction measurements to be performed on the mechanosynthesis of ZIFs (Halasz *et al.*, 2013 ▶), and the solvothermal synthesis of Cu-based MOFs has been investigated with the use of time-resolved PXRD techniques (Millange *et al.*, 2010 ▶, 2011 ▶), finding differences in the crystallization process and stability of the species formed during the reaction.

## 5. Concluding remarks   

The increasing complexity of MOF structures being reported is, at the moment, matched by advances in both crystallographic instrumentation and structural analysis techniques. Whilst large efforts are focused on obtaining single crystals of suitable diffraction quality, an increasing knowledge of powder diffraction data refinement can be used when this is not possible. The use of new methodologies for the analysis of powder diffraction data combined with advancements in structure computer modelling offer new ways to elucidate the crystal structures of MOFs. At the same time, reports on diffraction from MOFs under non-ambient conditions are increasing and provide much needed knowledge on their physical behaviour. Total scattering techniques are increasingly being applied for the study of MOF structures when conventional diffraction analysis is not sufficient, including the study of disordered materials or host–guest interactions.
